# A method for cell type marker discovery by high-throughput gene expression analysis of mixed cell populations

**DOI:** 10.1186/1472-6750-13-80

**Published:** 2013-10-03

**Authors:** Miguel A Andrade-Navarro, Femina Kanji, Carmen G Palii, Marjorie Brand, Harold Atkins, Carol Perez-Iratxeta

**Affiliations:** 1 Ottawa Hospital Research Institute, 501 Smyth Road, Ottawa, Ontario K1H 8L6, Canada; 2 Max Delbrück Center for Molecular Medicine, Robert-Rössle-Str. 10, 13125 Berlin, Germany; 3 Department of Cellular and Molecular Medicine, University of Ottawa, Ontario, K1H 8L6, Canada

**Keywords:** Cell markers, High-throughput gene expression, Cell isolation, Proof-of concept

## Abstract

**Background:**

Gene transcripts specifically expressed in a particular cell type (cell-type specific gene markers) are useful for its detection and isolation from a tissue or other cell mixtures. However, finding informative marker genes can be problematic when working with a poorly characterized cell type, as markers can only be unequivocally determined once the cell type has been isolated. We propose a method that could identify marker genes of an uncharacterized cell type within a mixed cell population, provided that the proportion of the cell type of interest in the mixture can be estimated by some indirect method, such as a functional assay.

**Results:**

We show that cell-type specific gene markers can be identified from the global gene expression of several cell mixtures that contain the cell type of interest in a known proportion by their high correlation to the concentration of the corresponding cell type across the mixtures.

**Conclusions:**

Genes detected using this high-throughput strategy would be candidate markers that may be useful in detecting or purifying a cell type from a particular biological context. We present an experimental proof-of-concept of this method using cell mixtures of various well-characterized hematopoietic cell types, and we evaluate the performance of the method in a benchmark that explores the requirements and range of validity of the approach.

## Background

Identification, isolation and characterization of individual cell types from mixed cell populations is a common problem in many fields of biomedicine including cell biology and clinical research, and has become especially important in cancer and stem cell research [1-3]. Cell-type specific gene markers allow the use of precise techniques for cell separation, such as fluorescence-activated cell sorting (FACS) [4], or for cell identification, for instance by polymerase chain reaction (PCR). However, it is problematic to identify marker genes when the cell type of interest is still poorly characterized because specifically expressed genes can only be unequivocally established once the cells have been isolated. Moreover, relying on inadequate markers could lead to incorrect conclusions about a target cell type, as experimental results based upon isolates supposedly containing only the pure target populations could be affected by the unbeknownst presence of other cell types.

**Figure 1:**
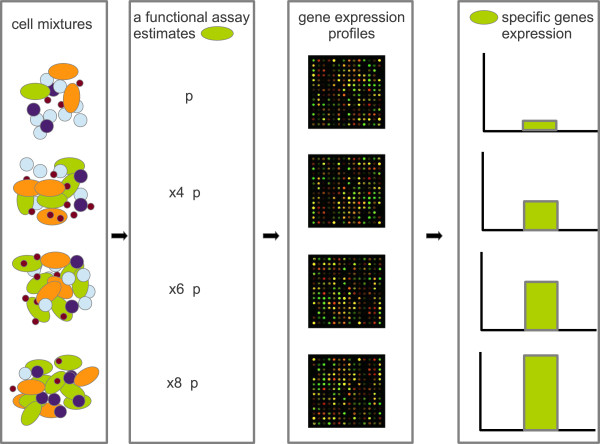
**Scheme of the proposed approach to obtain candidate markers starting from several cell mixtures that contain the cell type of interest and an estimate of its proportion in each mixture.** After gene expression profiling by a high-throughput technology, the expression of genes uniquely expressed in the cell type of interest should best correlate with the cell proportion estimates.

Here, we have tested the feasibility of a technique that employs high throughput gene expression analysis of cell mixtures to propose candidate genes as specific markers for a single cell-type. This strategy could be applied early in the characterization of a cell population as long as there is some indirect method, such as a *functional assay,* that estimates the concentration of the target cell type in the mixture. Given a mixture of cell types that contains the target cell type, our hypothesis is that the level of expression of specific transcripts (i.e. genes that the target cell type steadily expresses and no other cell type in the mixture appreciably produces), will be proportional to the amount of the target in the mixture. If a functional assay to estimate the relative amount of a cell type in a mixture exists, one could prepare a small number of samples with varying fractions of the target (see Figure 1). By high-throughput scans of gene expression on these samples, a set of markers could be hypothesized to be those whose gene expression best correlates to the estimated concentration of the target across the samples. It is expected that among the markers identified by this approach there will be a combination of specific cell-surface proteins that could be used in subsequent cell purification strategies.

This method is essentially an application of the simple assumption that there is a linear relation between cell type concentration in a population and its gene expression levels. This premise is straightforward and has been applied in the past for expression deconvolution of microarrays of heterogeneous cell samples for different applications [5-10], such as detecting cell-type specific differential expression between samples from mixed cell populations [7,10]. In our application to the problem of discovering new cell markers, we sought to determine whether this simple assumption could yield specific genes for an uncharacterized target cell-type mixed with an arbitrary number of cell types (characterized or not). Contrary to some previous studies (e.g. [10]), we will not be doing a full deconvolution of the mixtures because only the concentration of the target in the mixture is relevant to our purpose. We assumed that this proportion can be roughly estimated by some proxy, like a phenotypic property (e.g. the production of a metabolite) or a functional assay. Estimates for the composition or concentration of other cell types present in the mixture are not required.

We considered several factors that may affect the linearity of the association between marker expression and cell concentration. Some of these factors could potentially affect the actual expression of the genes by the target, such as cell-cell interactions between cell types within the mixture. Other influences would be related to technical aspects, particularly to the accuracy of gene expression measurement via a high-throughput technique. Very low or very high concentration levels of the cell type target could introduce non-linear effects in expression measurements by microarrays [11]. In addition, estimating the concentration of the cell type target in the mixtures through an indirect method is expected to carry some error.

Taking these considerations into account, there is still likely to be a sizable number of cell-type specific genes expressed in a linearly correlated manner across certain ranges of cell concentration. To test this hypothesis, we prepared five mixtures of four well characterized hematopoietic cell lines in known proportions and we profiled the gene expression of these mixtures by microarrays. Then we measured the correlation between the expression values of each gene in the microarrays and the proportion of each of the four cell types across the five mixtures. As a result, we were able to verify that genes whose expression was highly correlated to a particular cell-type concentration values were highly enriched for specific genes of that cell type, demonstrating the potential of this approach to the problem of specific marker gene discovery.

## Results and discussion

We hypothesized that genes that are specific to a given target cell type can be detected by analyzing a few samples of mixtures in which the target cell type has a varying concentration that can be estimated by a functional assay or some phenotypic feature. To evaluate the practical conditions under which this hypothesis may work, we prepared a total of five samples of mixed cell types by combining four leukemic cell lines: K562, HL60, Ly18 and Jurkat in varying proportions (see Methods and Table 1). To evaluate the limits imposed by cell abundance and level of gene expression, the cellular composition of the mixtures was designed to span wide and narrow ranges of cell target concentration, as well as large and small values of cell concentrations. The RNA extracted from these mixtures was analyzed on Affymetrix GeneChip Array HGU133 chips.

**Table 1:** Composition percentages of the four cell lines in the five mixtures

**Mixture**	**K562**	**HL60**	**Ly18**	**Jurkat**
**1**	0.1%	11.0%	15.0%	73.9%
**2**	1.0%	7.0%	24.0%	68.0%
**3**	3.0%	5.0%	30.0%	62.0%
**4**	2.0%	9.0%	35.0%	54.0%
**5**	0.3%	13.0%	40.0%	46.7%

To evaluate the performance of the method, we needed to compare the predicted marker candidates to a set of cell-type marker genes for each cell type. To this aim, we also profiled by microarray the gene expression of each of the four cell lines separately. Analyzing these pure cell type microrrays, we selected a set of specific genes for each of the four cell types. In this way, we defined gene markers for each cell line as those genes highly expressed only in that cell line (see *marker genes definition* in Methods). We also computed a *marker score* for each gene from the expression values in the pure samples (see *marker score definition* in Methods).

**Figure 2:**
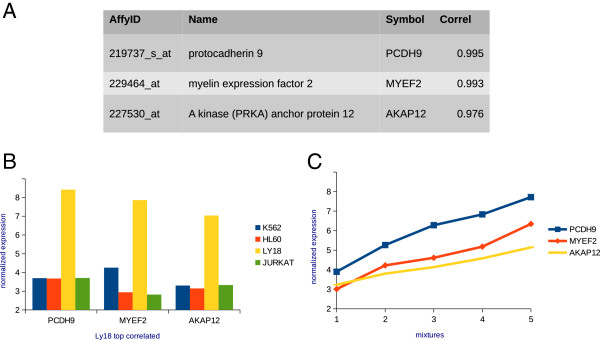
**Most correlated probesets to Ly18 concentration. (A)** Genes corresponding to the three most correlated probe sets to Ly18 cell line concentration. **(B)** Hybridization values in the four pure samples show that these probesets are only appreciably expressed in Ly18 cells. **(C)** Hybridization values in the mixtures versus the fraction of Ly18 show high correlation.

To measure the association of gene expression and cell type concentration for each of the four cell types, we computed the correlation by Pearson’s coefficient across the mixtures between the normalized intensity values of each microarray probe set and each cell type concentration. We included an additional data point corresponding to the origin, i.e. zero cell concentration and zero gene expression. To represent zero expression we used the global minimum of all normalized hybridization values across all the five mixture microarrays. The genes corresponding to the probe sets most correlated with a cell type concentration in the mixtures were compared to the list of markers for this cell type. These were previously defined from the pure cell arrays (see *marker genes definition* in Methods). As an example, Figure 2 shows the three topmost correlated probesets as the best candidate gene markers for Ly18 (Figure 2A-C) (see Additional file 1: Table S1). The three probesets are markers according to our definition (i.e. detected in the Ly18 pure sample and not detected in the other three pure cell type samples, Figure 2B), and could be considered specifically expressed genes of Ly18 in this particular context.

**Figure 3:**
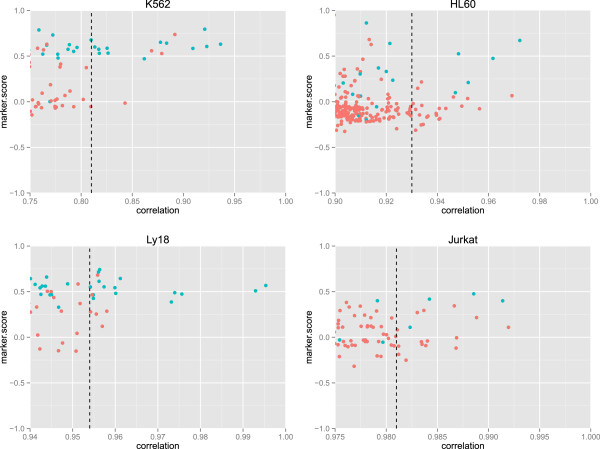
**Marker scores versus correlation coefficients for probe sets in each cell line.** High correlation coefficients correspond to high marker scores and often identify markers. Blue dots indicate markers, defined as present (P) in only that cell type, and orange dots are non-markers. The dashed lines indicate the value of correlation for the 20th most correlated probeset (see Table 2).

Our method performed generally well in predicting markers: for each of the four cell lines we detected a much higher number of positives among the top most correlated genes than would be expected by chance (see Table 2 and Additional file 2: Table S2). Interestingly, the worst performance corresponded to Jurkat, the cell line with the narrowest relative span in the range of concentrations among the samples; although even under this condition, more markers were detected amongst the best correlated genes than would be expected by chance (see Table 2). More unexpectedly, we could detect a high number of markers (with a large improvement over random) for the K562 line despite it being mixed in very low proportions. We hypothesize that a wide spread in the range of concentration could be helping this method to work well even if the absolute values of concentration are low. In general, we also observed that correlated genes have higher marker scores (see Figure 3 and full data in Additional file 2: Table S2). We verified that a small number of the markers deduced from the pure cell type arrays did have low marker score values. They correspond to specific genes with low level transcription (as detected by the microarrays). Hence, even if they are specifically expressed they might not be suitable markers.

**Table 2:** Performance of our method in detecting markers

**Cell line**	**Range**	**Markers**	**Top5**	**Top10**	**Top20**	**Expected top20**	**Correlation of 20th**
**K562**	0.1% to 3%	486	4	7	13	0 to 1 (0.523)	0.81
**HL60**	5% to 13%	1237	3	5	5	1 to 2 (1.30)	0.93
**Ly18**	15% to 40%	901	5	9	14	1 (0.95)	0.95
**Jurkat**	46.7% to 73.9%	792	2	3	4	1 (0.83)	0.98

range: range of fractions for the cell type in the mixtures. markers: number of markers defined as positives from the analysis of the pure samples and present at least in one of the mixtures; top5 (10, 20): number of positive markers (see Methods) among the 5 (10, 20) most correlated probesets; expected top20: expected numbers of markers if we randomly selected 20 probesets; correlation of 20th: value of the correlation coefficient for the 20th most correlated probe set.

**Figure 4:**
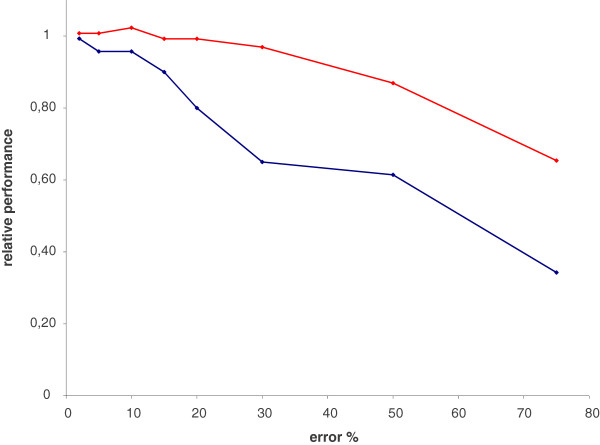
Relative performance (K562 in red, Ly18 in blue) decreases as error estimates of the target cell type concentration increase.

A necessary piece of information to apply this method is an estimate of the concentration of the target cell type in the mixtures. Hence, poor sensitivity of the functional assay in detecting differences of concentration of the target cell type is a potential limitation of this method. We wondered how errors in these estimates would affect the performance in terms of marker detection. Reasonably assuming that the errors would be of similar relative magnitude across all the mixtures, we measured how performance in marker detection degrades as errors in estimating the concentration of the target cell type in the mixtures grow. Errors in cell concentration estimates were simulated for each measure of the target cell type in the mixtures (random over and underestimations) from 2% to 75% of the actual cell concentration for two of the cell lines, K562 and Ly18. Figure 4 shows the average of the relative performance (n = 10) at each error level for both cell lines: K562 (red) and Ly18 (blue) (see also Additional file 3: Table S3). For errors below 20% performances stayed relatively close to the values for zero error estimate, which can be seen as substantial errors. For very high errors (over 20%) performance downgraded significantly.

Given the moderate cost of each array, we wondered how performance would downgrade if less than five mixtures were used in the analysis. The average performance was computed when dropping one or two samples from the analysis (i.e., average after removing all possible pairs) for the cell lines, K562 and Ly18. Using the datasets from only three mixtures resulted in a poor relative performance in detecting markers for both K562 and Ly18 (59% and 47% respectively). However, performance remained relatively high when using four samples (77% for K562 and 73% for Ly18, see Additional file 4: Table S4). Thus, these results indicate that four samples may be enough to detect marker genes amongst the top correlated genes. Accordingly, to put in practice this method, we recommend obtaining four, or preferably five or more cell mixtures, sampling variable concentrations of the target cell type. As four or five samples appear adequate to identify a number of markers and the arrays allow testing of thousands of genes at one time, the total cost of obtaining markers would be less than the cost of testing a large number of individual potential markers. After gene expression analysis of the samples, marker genes can be selected according to the correlation of their expression to cell concentration. It is advisable to verify that potential markers are considered from the top most correlated genes, that they have very high values of correlation, and that some of the functions of the corresponding transcripts are consistent with the cell type of interest. If the selected markers are inadequate, it is possible to prepare one or two additional mixtures to obtain better correlation values and markers.

A simple strategy to produce samples with a wide range of cell concentrations would be to deplete or enrich known cell types in the samples. In our experiment, we have used mixtures with uncorrelated values of concentration for all the four cell types. One can guess that in the hypothetical situation in which a group of two or more cell types happens to be correlated across the samples, the chances of obtaining markers will be necessarily lower than as presented here. However, even in this situation the detection of markers for a subset of associated cells might be useful and lead eventually to their individual characterization.

Common approaches for processing microarray data seem adequate for our approach. Alternative methods for normalizing array intensities did not improve the overall performance in terms of number of detected markers (data not shown). Furthermore, this analytical method for the identification of markers could be applied to quantitative gene expression datasets obtained from more recently developed alternatives to microarrays, like full transcriptome sequencing. However, in the near future, microarrays could remain the most advantageous cost-wise.

We expect that the sensitivity of the high-throughput technique used to measure the RNA levels sets a lower limit to the concentration of a cell type that could be successfully analyzed with this technique. For microarrays, this limit is given by the smallest fraction of mRNA that can be reliably detected by hybridization to the cognate DNA. It matches the sensitivity of other techniques of RNA detection (i.e., northern blot) and is estimated to be approximately 1–2 copies per million of mRNA molecules [12]. Our proposed method could work even for cases where the cells of interest are present in relatively low concentrations (e.g. less than 3%) provided that the range of concentrations is sufficiently wide. Looking at the number of markers for K562 detected in our experiment – the rarest cell line in the samples - we extrapolate that just 35 or fewer markers would be detect for a cell type present at a concentration of 0.01% (see Additional file 5: Table S5). Although these should correspond to highly expressed transcripts, and hence reliable markers, the relative low number of markers indicates that this method could not be applied at lower cell concentrations unless some enriching protocol can be previously carried out on the mixtures. This enrichment could be achieved by depleting a characterized cell type known to be present in the mixtures. Thus, our strategy could be useful for detecting markers of rare cells in a population, which suggests that it could be particularly useful as a method to identify candidate markers of stem/progenitor cell populations that are notoriously difficult to isolate as pure populations.

Our method is dependent on functional tests that must be both highly sensitive and quantitative. It is important to note that functional assays that display these two characteristics have been designed and are routinely used by laboratories studying particular types of stem/progenitor cells. For example, hematopoietic stem cells are effectively detected and quantified at the single cell level using both *in vitro* (e.g. Long-Term Culture-Initiating Cell (LTC-IC) Assay) and *in vivo* (e.g. engraftment into immunodeficient mice) approaches [13]. Another example is represented by endothelial stem/progenitors cells. Indeed, while these cells can be readily identified and quantified by colony assays similar to those used for quantification of hematopoietic stem cells, their function can be quantified through assays that enumerate the vascular network these cells form *in vitro* when they are plated on a Matrigel matrix or *in vivo* when they are transplanted into immunodeficient mice [14]. While the above examples relate to the hemato-endothelial lineages, it is important to note that most of the laboratories that specialize in specific types of stem/progenitor cells have developed highly sensitive and quantitative functional assays targeted to their cell type of interest. Thus our method should be highly adaptable to these different cell types.

## Conclusion

Cell-type specific gene markers will continue to play a major role in advancing research in many areas of the life-sciences, particularly in stem cell biology. As new types of stem and progenitor cells are identified and their functions are established, identifying specific markers will facilitate their use in experimental systems. Specific gene markers or combinations thereof remain to be identified for endothelial stem/progenitor cells among many other cell types [15]. This method could facilitate the specific identification, purification and characterization of such cell types.

## Methods

### Cell mixtures microarray analyses

We prepared a total of five samples of cell mixtures from four different hematopoietic cell lines K562, HL60, Ly18 and Jurkat (see compositions in Table 1). The concentration of each cell line was measured using a Coulter counter prior to mixing. Defined volumes of the cell line stocks were mixed to give the desired ratio of the cell lines in the mixed samples. K562, HL60 and Jurkat cells were purchased from ATCC (Manassas, Virginia, US). Ly18 cells (OCI-Ly18 in [16]) were a gift from Dr. Hans Messner from the University Health Network in Toronto. The gene expression profile of each of the five mixtures was analyzed on Affymetrix GeneChip Array HGU133 chips (five arrays). We also profiled the expression of the four cell lines separately (four arrays). Hybridization values were normalized with GCRMA [17].

### Marker gene definitions

To evaluate our approach to detect marker genes in cell mixtures we required the definition of a set of *marker genes* for each cell type, i.e., genes highly expressed in that cell type and not appreciably expressed in the rest. To define these sets we used the microarray of the four pure cell types. Software MAS5.0 calls every probeset in an array as either absent (A), present (P), or marginal (M) (i.e., undecided). Accordingly, we defined markers of a given cell type as those genes with probesets that are P in the corresponding pure cell type microarray, and either A or M in the other three. This classifies the genes into markers and non-markers. All probe sets with absent calls for every mixture were filtered out from subsequent analyses as corresponding to non-expressed transcripts.

### Marker score definitions

To have a quantitative measurement in addition to the maker definition above, we defined a *marker score,* as a number that describes how well a probeset in the microarray could perform as marker of a given cell type. The marker score was defined as the product of the normalized hybridization value of the probeset for the pure cell type by the z-score of the distribution of hybridization values of the probeset for the four pure cell types. Thus, genes that are highly expressed in a cell type and lowly expressed in the other three will have large values of the marker score for the cell type in which they are highly expressed.

## Competing interests

The authors declare no competing interests.

## Authors’ contributions

MA-N, HA and CP-I devised the method and designed the study. FK, CGP and MB carried on the experimental work. CP-I analyzed the data. MA-N and CP-I wrote the manuscript. All authors contributed to the manuscript. All authors read and approved the final manuscript.

## Supplementary Material

Additional file 1: Table S1 Top 10 most correlated probesets to Ly18. Click here for file

Additional file 2: Table S2 Correlation of probesets with proportion of cell type in the mixtures, marker scores and whether the probesets are mapped to markers or not. Contains four sheets, one per cell line. Click here for file

Additional file 3: Table S3 Number of markers detected and relative performance for cell lines Ly18 and K562 for different error values in the proportion of cell type estimates. Click here for file

Additional file 4: Table S4 Number of markers detected and relative performance for cell lines Ly18 and K562 when using three or four mixtures. Click here for file

Additional file 5: Table S5 Number of markers of K562 that are detected as a function of the proportion of K562 cells in a mixture. Click here for file

## References

[B1] O’Brien CA Pollett A Gallinger S Dick JE A human colon cancer cell capable of initiating tumour growth in immunodeficient mice Nature 2007 445 106 110 10.1038/nature05372 17122772

[B2] Ramirez JM Gerbal-Chaloin S Milhavet O Qiang B Becker F Assou S Lemaître JM Hamamah S De Vos J Brief report: benchmarking human pluripotent stem cell markers during differentiation into the three germ layers unveils a striking heterogeneity: all markers are not equal Stem Cells 2011 29 1469 1474 21714037 10.1002/stem.681

[B3] Stewart MH Bendall SC Bhatia M Deconstructing human embryonic stem cell cultures: niche regulation of self-renewal and pluripotency J Mol Med 2008 86 875 886 10.1007/s00109-008-0356-9 18521556

[B4] Herzenberg LA De Rosa SC Monoclonal antibodies and the FACS: complementary tools for immunobiology and medicine Immunol Today 2000 21 383 390 10.1016/S0167-5699(00)01678-9 10916141

[B5] Venet D Pecasse F Maenhaut C Bersini H Separation of samples into their constituents using gene expression data Bioinformatics 2001 17 Suppl 1 S279 S287 10.1093/bioinformatics/17.suppl_1.S279 11473019

[B6] Lu P Nakorchevskiy A Marcotte EM Expression deconvolution: a reinterpretation of DNA microarray data reveals dynamic changes in cell populations Proc Natl Acad Sci U S A 2003 100 10370 10375 10.1073/pnas.1832361100 12934019 PMC193568

[B7] Ghosh D Mixture models for assessing differential expression in complex tissues using microarray data Bioinformatics 2004 20 1663 1669 10.1093/bioinformatics/bth139 14988124

[B8] Lähdesmäki H Shmulevich L Dunmire V Yli-Harja O Zhang W In silico microdissection of microarray data from heterogeneous cell populations BMC Bioinforma 2005 6 54 10.1186/1471-2105-6-54 PMC1274251 15766384

[B9] Repsilber D Kern S Telaar A Walzl G Black GF Selbig J Parida SK Kaufmann SH Jacobsen M Biomarker discovery in heterogeneous tissue samples -taking the in-silico deconfounding approach BMC Bioinforma 2010 11 27 10.1186/1471-2105-11-27 PMC3098067 20070912

[B10] Shen-Orr SS Tibshirani R Khatri P Bodian DL Staedtler F Perry NM Hastie T Sarwal MM Davis MM Butte AJ Cell type-specific gene expression differences in complex tissues Nat Methods 2010 7 287 289 10.1038/nmeth.1439 20208531 PMC3699332

[B11] Halperin A Buhot A Zhulina EB Sensitivity, specificity, and the hybridization isotherms of DNA chips Biophys J 2004 86 718 730 10.1016/S0006-3495(04)74150-8 14747310 PMC1303922

[B12] Bittner M Butow R DeRisi J Diehn M Eberwine J Epstein CB Glynne R Grimmond S Ideker T Kacharmina JE Katsabanis S Khan J Lee J Liu CL Marciano P Marincola FM McIntosh T Monte D Pollack JR Rhodius V Somerville S Tom E Wang E Wei JS Willhite D Ybarra S Bowtell D, Sambrook J Expression Analysis of RNA DNA Microarrays: a molecular cloning manual 2003 Cold Spring Harbor, NY: Cold Spring Harbor Laboratory Press 101 288

[B13] Notta F Doulatov S Laurenti E Poeppl A Jurisica I Dick JE Isolation of single human hematopoietic stem cells capable of long-term multilineage engraftment Science 2011 333 218 221 10.1126/science.1201219 21737740

[B14] Yoder MC Defining human endothelial progenitor cells J Thromb Haemost 2009 7 Suppl 1 49 52 19630767 10.1111/j.1538-7836.2009.03407.x

[B15] Rizk P Barker N Gut stem cells in tissue renewal and disease: methods, markers, and myths Wiley Interdiscip Rev Syst Biol Med 2012 4 475 496 10.1002/wsbm.1176 22644962

[B16] Chang H Leeder S Cook VA Patterson B Dosch M Minden MD Messner HA Growth of human lymphoma cells in SCID mice Leuk Lymphoma 1992 8 129 10.3109/10428199209049826 1493464

[B17] Hubbell E Liu WM Mei R Robust estimators for expression analysis Bioinformatics 2002 18 1585 1592 10.1093/bioinformatics/18.12.1585 12490442

